# Reviving the African Wolf *Canis lupus lupaster* in North and West Africa: A Mitochondrial Lineage Ranging More than 6,000 km Wide

**DOI:** 10.1371/journal.pone.0042740

**Published:** 2012-08-10

**Authors:** Philippe Gaubert, Cécile Bloch, Slim Benyacoub, Adnan Abdelhamid, Paolo Pagani, Chabi Adéyèmi Marc Sylvestre Djagoun, Arnaud Couloux, Sylvain Dufour

**Affiliations:** 1 UMR BOREA IRD 207, Muséum National d’Histoire Naturelle, Paris, France; 2 SYLVATROP, Nantes, France; 3 Laboratoire d’Ecologie des Systèmes Terrestres et Aquatiques, Faculté des Sciences, Université d’Annaba, Algérie; 4 Egyptian Environmental Affairs Agency, Nature Conservation Sector, Cairo, Egypt; 5 Dutch Wildlife Health Centre, Utrecht, The Netherlands; 6 Laboratoire d’Ecologie Appliquée, Faculté des Sciences Agronomiques, Université d’Abomey-Calavi, Cotonou, Benin; 7 Genoscope, Centre National de Séquençage, Evry, France; Barnard College, Columbia University, United States of America

## Abstract

The recent discovery of a lineage of gray wolf in North-East Africa suggests the presence of a cryptic canid on the continent, the African wolf *Canis lupus lupaster*. We analyzed the mtDNA diversity (cytochrome *b* and control region) of a series of African *Canis* including wolf-like animals from North and West Africa. Our objectives were to assess the actual range of *C. l. lupaster*, to further estimate the genetic characteristics and demographic history of its lineage, and to question its taxonomic delineation from the golden jackal *C. aureus*, with which it has been considered synonymous. We confirmed the existence of four distinct lineages within the gray wolf, including *C. lupus/familiaris* (Holarctic wolves and dogs), *C. l. pallipes*, *C. l. chanco* and *C. l. lupaster*. Taxonomic assignment procedures identified wolf-like individuals from Algeria, Mali and Senegal, as belonging to *C. l. lupaster*, expanding its known distribution c. 6,000 km to the west. We estimated that the African wolf lineage (i) had the highest level of genetic diversity within *C. lupus*, (ii) coalesced during the Late Pleistocene, contemporaneously with Holarctic wolves and dogs, and (iii) had an effective population size of c. 80,000 females. Our results suggest that the African wolf is a relatively ancient gray wolf lineage with a fairly large, past effective population size, as also suggested by the Pleistocene fossil record. Unique field observations in Senegal allowed us to provide a morphological and behavioral diagnosis of the African wolf that clearly distinguished it from the sympatric golden jackal. However, the detection of *C. l. lupaster* mtDNA haplotypes in *C. aureus* from Senegal brings the delineation between the African wolf and the golden jackal into question. In terms of conservation, it appears urgent to further characterize the status of the African wolf with regard to the African golden jackal.

## Introduction

The gray wolf (*Canis lupus* Linnaeus, 1758) is one of the most emblematic, extant mammalian species: once the most widely distributed mammal–encompassing a Holarctic and Indian subcontinent distribution [1], it was domesticated to become “Man’s best friend”, the dog [2]. Nevertheless, the general aversion of public opinion towards the gray wolf has led to its complete extirpation from Mexico, most of United States and Western Europe, and many populations throughout the world are threatened by human competition for livestock and game, and habitat fragmentation [3].

Despite the delineation of at least 30 subspecies [4] and a noticeable phenotypic variation (in body size and coat pattern) throughout its range, the gray wolf has been considered a species with relatively weak morphometric and genetic structuring [5], [6]. However, recent investigations focusing on understudied areas of the species’ range identified two distinct mitochondrial lineages from India and the Himalayan-Tibetan plateau (attributed to the subspecies *pallipes* Sykes, 1831 and *chanco* Gray, 1863, respectively) [7], [8], which were most likely not involved in dog domestication.

The gray wolf is generally not considered to occur in Africa (reaching the Sinai Peninsula, northeastern Egypt; [3]), where it is ecologically ‘replaced’ by the golden jackal (*Canis aureus* Linnaeus, 1758), which itself ranges from the northern half of Africa to south-eastern Europe and Asia. However, it has long been emphasized that the so-called Egyptian jackal *C. aureus lupaster* Hemprich & Ehrenberg, 1832, distributed in North Africa, had cranial and dental sizes that overlapped with the smaller-sized wolves from Arabia and India, but clearly separated from the even smaller golden jackal [9], [10], [11], [12]. On these morphological grounds, Ferguson [9] proposed to consider *lupaster* as a subspecies of gray wolf, with a distribution restricted to Egypt and Libya. Supporting this view, two recent studies detected a divergent mitochondrial lineage of gray wolf in northern Egypt and Ethiopia [13], [14] that was eventually designated as the African wolf *C. lupus lupaster* [13]. Wolves (from Ethiopia) were larger–but slender-looking–than the usual golden jackal phenotype, expanding the gray wolf’s range more than 2500 km south-east into the African continent.

**Figure 1:**
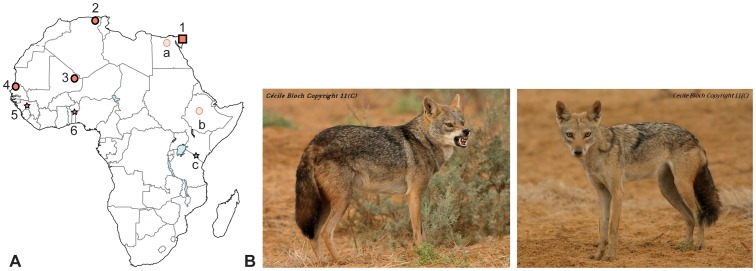
**A- Geographic distribution of the African** ***Canis*** **included in this study.** Numbers indicate samples collected for this study (see Table 1), and letters refer to the nucleotide sequences available in the literature or Genbank for cytochrome *b*. Square, circles and stars represent mtDNA-typed *Canis lupus/familiaris*, *C. l. lupaster*, and *Canis* spp. other than *C. lupus*, respectively: 1- near Sharm el Shikh city, south Sinai, Egypt; 2- coastal region between Skikda and El-Kala, Algeria; 3- Terarabat, Adrar des Iforas, Mali; 4- near Kheune, east of P.N. du Djoudj, Senegal; 5- Koubia, Dalaba and Tougue districts, Guinea; 6- Béterou, Benin; a- northern Egypt [14]; b- Menz region, Ethiopia [13]; c- southern Kenya and northern Tanzania [53]. **B- The typical ‘wolf-like’ (left) and ‘jackal-like’ (right) phenotypes observed near Kheune, Senegal.** (photographs: C. Bloch).

The discovery of a distinct lineage endemic to Africa of such a flagship species as the gray wolf is critical in terms of conservation, especially since large African canids do not benefit from any specific conservation action, and are regularly persecuted to protect livestock [15], [16]. This discovery also raises a series of overlapping questions. First, how could a gray wolf lineage have passed undetected in Africa until recently? And how long and how far has it been ranging the continent? Feeding the debate, large forms of ‘jackals’ comparable to *lupaster* have been reported from the Middle to Late Pleistocene of Morocco [17]. Second, how can the African phenotype of the gray wolf be defined? In other words, is there a clear phenotypic distinction between the gray wolf and the golden jackal, the latter also showing a wide spectrum of morphological and ecological variations throughout its distribution [18]? And third, does the gray wolf’s African phenotype reflect adaptation to specific environmental conditions or rather result from potential hybridization with the golden jackal? Although no crosses between the two species have been reported to date, hybridization among *Canis* taxa has proved to be common [19], [20], [21] and to involve significant phenotypic changes in hybrid generations [22], reaching fixation in several cases [23], [24].

**Table 1:** List of the African canids sampled for this study, with their taxonomic assignment based on mitochondrial DNA sequences.

Taxa	Sample number	Origin	Genbank accession number		MtDNA-based assignment	
			*CYTB*	CR	*CYTB*	CR
					(SAP assignment/level of identity)	
**African wolves and jackals**						
*Canis lupus*	T1333	Egypt, south Sinai, north of Sharm elShikh city	JQ088658	JQ088677	*Canis lupus/familiaris*(49%/100%)	*Canis lupus/familiaris*(49%/100%)
*Canis* sp.	T1361	Senegal, 12 km east of Parc Nationaldu Djoudj, near Kheune	JQ088664	JQ088683	*Canis lupus lupaster*(69%/99%)	*Canis lupus lupaster*(92%/95%)
*Canis* sp.	T1341	Algeria, coastal region between Skikdaand El-Kala	JQ088659	JQ088678	*Canis lupus lupaster*(63%/99%)	*Canis lupus lupaster*(55%/95%)
*Canis* sp.	T1346	Algeria, coastal region between Skikdaand El-Kala	JQ088660	JQ088679	*Canis lupus lupaster*(69%/99%)	*Canis lupus lupaster*(50%/95%)
*Canis* sp.	T1347	Algeria, coastal region between Skikdaand El-Kala	JQ088661	JQ088680	*Canis lupus lupaster*(62%/96%)	*Canis lupus lupaster*(82%/95%)
*Canis* sp.	T1348	Algeria, coastal region between Skikdaand El-Kala	JQ088662	JQ088681	*Canis lupus lupaster*(69%/99%)	*Canis lupus lupaster*(58%/95%)
*Canis* sp.	T1349	Algeria, coastal region between Skikdaand El-Kala	JQ088663	JQ088682	*Canis lupus lupaster*(69%/99%)	*Canis lupus lupaster*(66%/95%)
Canis sp.	T1621*	Mali, Adrar des Iforas, Terarabat	JQ088665	JQ088684	*Canis lupus lupaster*(75%/99%)	*Canis lupus lupaster*(57%/95%)
*Canis aureus*	T1360	Senegal, 12 km east of Parc Nationaldu Djoudj, near Kheune	JQ088656	JQ088675	*Canis lupus lupaster*(61%/99%)	*Canis lupus lupaster*(61%/95%)
*Canis aureus*	T1362	Senegal, Dakar, Zoo du Parc de Hann(fourth captive generation)	JQ088657	JQ088676	*Canis lupus lupaster*(61%/99%)	*Canis lupus lupaster*(61%/95%)
*Canis adustus*	T1250	Guinea, Koubia, Pilimini	JQ088650	JQ088669	NA	NA
*Canis adustus*	T1252	Guinea, Koubia, Pilimini	JQ088651	JQ088670	NA	NA
*Canis adustus*	T1257	Guinea, Dalaba, Dalaba cen.	JQ088652	JQ088671	NA	NA
*Canis adustus*	T1262	Guinea, Tougue, Kollet	JQ088653	JQ088672	NA	NA
*Canis adustus*	T1263	Guinea, Tougue, Kollet	JQ088654	JQ088673	NA	NA
*Canis adustus*	T806	Benin, Béterou	JQ088655	JQ088674	NA	NA

Assignment was based on cytochrome *b* (*CYTB*) and control region (CR), using the Statistical Assignment Package (see Materials and Methods).

* MNHN tissue bank: n° 1999-152.

NA, reliable set of homologues could not be compiled.

In this study, we analyzed the mitochondrial DNA (mtDNA) diversity of a series of African *Canis* including wolf-like animals from North and West Africa (Fig. 1), to respond to the following questions: (i) is *C. lupus lupaster* confined to Egypt and Ethiopia?, (ii) does it constitute an ancient African lineage or a recent spread into the continent?, and (iii) does hybridization between the African wolf and the golden jackal occur? Our results suggest that (i) the distribution of the African wolf also includes North and West Africa, expanding its range 6,000 km to the west; (ii) *C. l. lupaster* is a distinct, relatively ancient and genetically highly diversified lineage of gray wolf endemic to Africa; and (iii) hybridization between the former and *C. aureus* may occur in West Africa, although the ‘golden jackal’ entity needs to be reassessed further. We also provide unique information on the morphology and behavior of sympatric African wolves and golden jackals from West Africa. We expect that further taxonomic characterization of the African wolf, notably using the data obtained in this study, could supplement the rapid establishment of a targeted conservation strategy.

**Figure 2:**
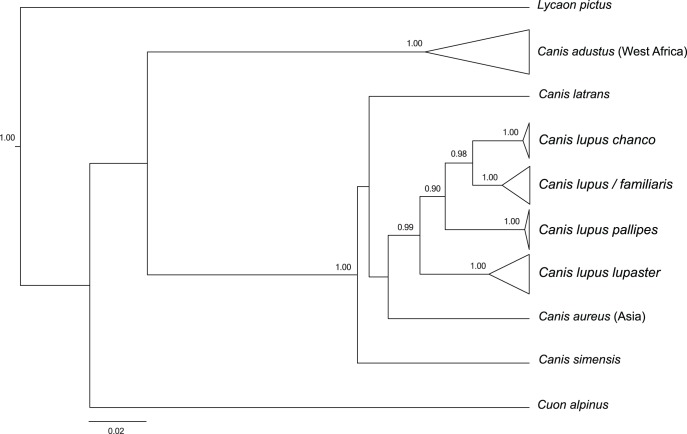
Bayesian phylogenetic analysis of the wolf-like clade based on cytochrome *b* and control region. The model HKY + I + Γ was applied to the coherent fragment “cytochrome *b* + control region”, assuming a constant size coalescent model. Values at nodes correspond to posterior probabilities ≥0.90. Clades were collapsed for better readability of the tree. Scale bar represents 2% sequence divergence.

## Results

The Statistical Assignment Package (SAP) procedure identified the seven *Canis* sp. from Algeria, Mali and Senegal, and the two *C. aureus* from Senegal, as belonging to the African wolf lineage *C. l. lupaster* (Table 1). Although the level of confidence in the assignment varied (from 50 to 82%) using either cytochrome *b* (*CYTB*) or control region (CR) fragments, the highest match (95–99%) was always found with the *C. l. lupaster* sequences registered in Genbank. The individual from Egypt was assigned to *C. lupus/familiaris* (SAP: 49%; level of similarity: 100%). Reliable sets of homologues (i.e. with minimum identity ≥0.9) could not be compiled for the sequences of West African *C. adustus* that we generated.

**Figure 3:**
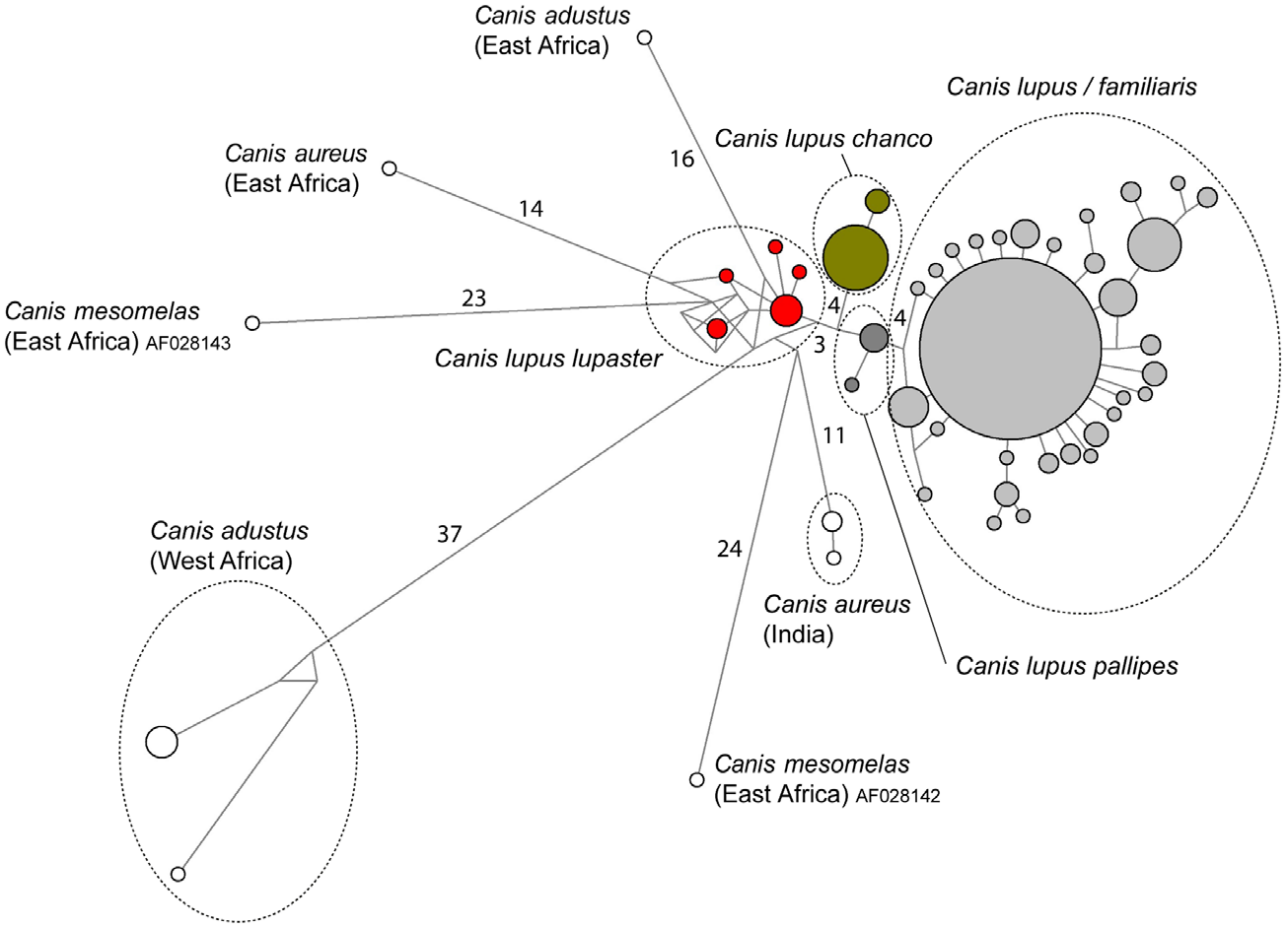
Median-joining networks of *Canis lupus* and jackal haplotypes based on cytochrome *b*. Circle size and branches are proportional to haplotype frequency and number of mutation steps among haplotypes, respectively. Numbers refer to mutation steps separating the main haplogroups (circled). To improve overall visualization of the network, branch proportions were not respected within the *Canis lupus/familiaris* haplogroup.

The monophyly of the gray wolf clade (*Canis lupus* spp.) was strongly supported (posterior probability pp = 1; Fig. 2). This clade was included in a monophyletic group (pp = 1) composed of *C. simensis, C. aureus* (from Asia) and *C. latrans*. Within the *C. lupus* clade, the four lineages were recovered monophyletic (pp = 1), and branched as (*lupaster*, (*pallipes*, (*lupus/familiaris*, *chanco*))).

**Table 2:** Mean genetic distances (below diagonal) among the main *Canis lupus* lineages and the other species of the wolf-like clade.

	Taxon	1	2	3	4	5	6	7	8	9	10	11	12	13	14
1	*Canis lupus/familiaris*		0.011	0.007	0.010	0.015	0.016	0.016	0.014	0.019	0.026	0.025	0.018	0.020	0.022
2	*C. l. lupaster*	0.043		0.009	0.007	0.012	0.013	0.012	0.012	0.018	0.023	0.022	0.013	0.019	0.018
3	*C. l. pallipes*	0.018	0.025		0.007	0.012	0.014	0.014	0.011	0.018	0.024	0.024	0.016	0.019	0.019
4	*C. l. chanco*	0.032	0.019	0.014		0.012	0.013	0.012	0.011	0.017	0.024	0.022	0.014	0.018	0.019
5	*C. latrans*	0.066	0.047	0.048	0.042		0.016	0.013	0.012	0.019	0.024	0.024	0.017	0.019	0.020
6	*C. aureus* (East Africa)	0.079	0.054	0.060	0.052	0.072		0.015	0.016	0.020	0.022	0.022	0.014	0.021	0.017
7	*C. aureus* (India)	0.072	0.045	0.053	0.046	0.052	0.064		0.013	0.020	0.024	0.022	0.014	0.018	0.021
8	*C. simensis*	0.061	0.045	0.042	0.041	0.041	0.083	0.047		0.019	0.024	0.021	0.016	0.017	0.018
9	*Cuon alpinus*	0.100	0.092	0.089	0.089	0.102	0.109	0.108	0.107		0.019	0.021	0.019	0.021	0.021
10	*Lycaon pictus*	0.157	0.130	0.136	0.135	0.140	0.130	0.143	0.140	0.105		0.025	0.022	0.023	0.023
11	*C. adustus* (West Africa)	0.160	0.136	0.148	0.140	0.147	0.141	0.143	0.124	0.131	0.144		0.022	0.023	0.022
12	*C. adustus* (East Africa)	0.098	0.058	0.079	0.063	0.083	0.066	0.064	0.079	0.105	0.130	0.135		0.020	0.021
13	*C. mesomelas* (AF028142)	0.110	0.093	0.099	0.090	0.102	0.105	0.095	0.083	0.117	0.122	0.140	0.109		0.020
14	*C. mesomelas* (AF028143)	0.117	0.089	0.099	0.098	0.109	0.085	0.111	0.091	0.113	0.125	0.129	0.121	0.093	

Genetic distances were estimated from cytochrome *b* using the Kimura 2-parameter model. Standard error estimates are given above diagonal.

The *CYTB* network analyses distinguished the four above-mentioned lineages within *Canis lupus* (*C. lupus/familiaris*, *C. l. pallipes, C. l. chanco* and *C. l. lupaster*; Fig. 3). Mutation steps between connecting pairs of haplogroups varied between 3 to 4. Haplogroups representing jackals were distant from wolf lineages by 11 to 24 mutation steps. None of the *CYTB* sequences representing the jackal species *C. aureus, C. adustus* and *C. mesomelas*, grouped into single clusters.

**Table 3:** Genetic diversity in the main mtDNA lineages found in *Canis lupus*.

Taxa (n individuals *CYTB*/CR)	*S*		π		H		Hd	
	*CYTB*	CR	*CYTB*	CR	*CYTB*	CR	*CYTB*	CR
*Canis lupus/familiaris*	32	72	0.00243 (0.00028)	0.01769 (NA)	31	150	0.424 (0.037)	0.923 (0.0036)
n = 292/n = 1382								
*Canis l. pallipes*	1	2	0.00130 (0.00077)	0.00291 (0.00173)	2	2	0.400 (0.237)	0.400 (0.237)
n = 5/n = 5								
*Canis l. chanco*	1	3	0.00077 (0.00034)	0.00345 (0.00118)	2	2	0.237 (0.105)	0.312 (0.106)
n = 23/n = 22								
*Canis l. lupaster*	6	22	0.00492 (0.00129)	0.02795 (0.00445)	5	9	0.756 (0.130)	0.978 (0.054)
n = 10/n = 10								

Standard deviation is given between parentheses. *S*, number of polymorphic sites; π, nucleotide diversity; h, number of haplotypes; Hd, haplotype diversity.

Mean *CYTB* K2P distances among *C. lupus/familiaris* and the three other wolf lineages varied between 0.018 (*C. l. pallipes*), 0.032 (*C. l. chanco*) and 0.043 (*C. l. lupaster*) (Table 2). The closest taxa to *C. lupus* were *C. simensis* and *C. latrans* (0.041 to 0.061 and 0.042 to 0.066, respectively), whereas *C. adustus* from West Africa and *L. pictus* were the most distant (0.136 to 0.160 and 0.130 to 0.157, respectively). Genetic distances between Indian and East African *C. aureus*, West and East African *C. adustus*, and East African *C. mesomelas* were 0.064, 0.135 and 0.093, respectively.

**Table 4:** Time to most recent common ancestor (TMRCA) in the four *Canis lupus* lineages.

Wolf lineage	TMRCA (yrs)	95% HPD
*C. l. lupus/familiaris*	232,000	139,000–366,000
*C. l. pallipes*	23,000	3,000–60,000
*C. l. chanco*	22,000	2,000–58,000
*C. l. lupaster*	288,000	152,000–494,000

HPD: Highest Posterior Density.

Among the four wolf lineages, *C. l. lupaster* showed the highest nucleotide and haplotype diversity (*S* = 0.00492–0.02795; Hd = 0.756–0.978 – for *CYTB* and CR, respectively), followed by *C. lupus/familiaris* (*S* = 0.00243–0.01769; Hd = 0.424–0.923); *C. l. pallipes* and *C. l. chanco* had dramatically lower genetic diversity estimates (Table 3).

**Figure 4:**
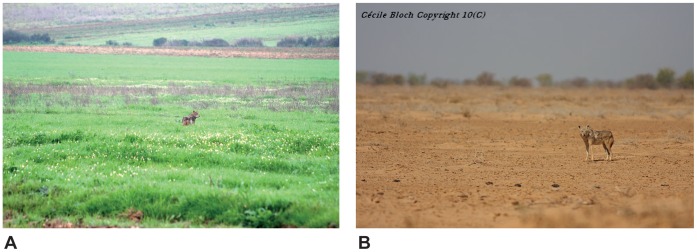
Habitats of the African wolf. A- Agricultural landscape with wildland (*Asphodelus microcarpus*), coastal region between Skikda and El-Kala, Algeria (photograph: S. Benyacoub); B- Sandy Sahelian savannah with scarce shrub cover (*Tamarix senegalensis*), near Kheune, Senegal (photograph: C. Bloch).

Time to most recent common ancestor (TMRCA) as estimated with BEAST yielded similar values for *C. l. lupaster* and *C. l. lupus/familiaris* (median = 288,000 and 232,000 years, respectively), which was c. 10-fold greater than for the Indian and Himalayan wolf lineages (Table 4).

**Table 5:** Female effective population size (N_ef_) in the four *Canis lupus* lineages.

Wolf lineage	N_ef_	95% CI
*C. l. lupus/familiaris*	105,000	73,000–148,000
*C. l. pallipes*	6,500	1,200–32,000
*C. l. chanco*	4,000	1,300–11,000
*C. l. lupaster*	80,000	39,000–182,000

Confidence intervals (CI) were calculated from the percentage profile likelihoods in LAMARC (see Materials and Methods).

*Canis l. lupaster* and *C. l. lupus/familiaris* had the greatest female effective population sizes (N_ef_ = 80,000 and 105,000, respectively), whereas the Indian and Himalayan wolf lineages had N_ef_ <7,000 individuals.

**Figure 5:**
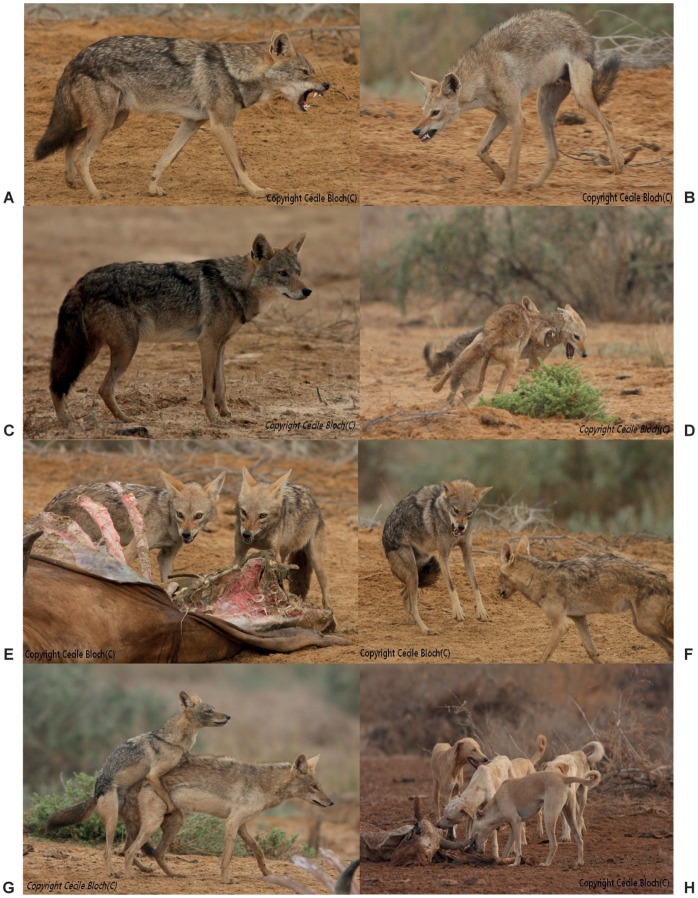
Phenotypic and behavioral traits between *Canis* species near Kheune, Senegal. A- Aggressive posture of the African wolf towards golden jackals; B- Aggressive posture of a golden jackal towards congeners; C- Typical ‘wolf-like’ phenotype, sampled in this study (T1361); D- Typical ‘jackal-like’ phenotypes, sampled in this study (T1360); E- Food guarding of golden jackals on a dead carcass of cow; E- African wolf (left) fighting with golden jackal (right) to access the dead carcass; F- Simulated mating between two male golden jackals; G- Phenotype of the feral dogs living in sympatry with the African wolf and the golden jackal. (photographs: C. Bloch).

## Discussion

Our mitochondrial phylogenetic analysis confirmed the existence of four distinct lineages within the gray wolf, including *C. lupus/familiaris* (Holarctic wolves and dogs), *C. l. chanco* (Himalayan wolf), *C. l. pallipes* (Indian wolf) and *C. l. lupaster* (African wolf) (Fig. 2).

**Figure 6:**
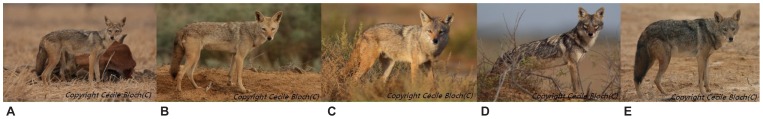
Phenotypic variation in the golden jackal and the African wolf near Kheune, Senegal. A- Typical ‘jackal-like’ phenotype; B- ‘Jackal-like’ phenotype tending towards C; C- ‘Intermediate’ phenotype between golden jackal and African wolf; D- ‘wolf-like’ phenotype tending towards C; E- Typical ‘wolf-like’ phenotype. (photographs: C. Bloch).

The taxonomic assignment procedure (SAP) identified the *Canis* sp. from Algeria, Mali and Senegal, as belonging to the African wolf mtDNA lineage *C. l. lupaster* (Table 1). Variation in the SAP assignment confidence estimates reflected the uncertain relationships among gray wolf lineages, but the Bayesian phylogenetic analysis, the haplogroups defined by the network analysis and the observed high levels of similarity with the Genbank sequences of African wolf confirmed the association of the newly generated sequences to the latter lineage (Figs. 2 and 3, Table 1). Thus, our results expand the distribution of the African wolf in North and West Africa, more than 6,000 km west from its previously determined range in North-East Africa [9], [13] (Fig. 1). This contradicts the craniometric analyses of Krystufek & Tcrtkovic [12], whom found that ‘*lupaster*’ from Egypt and Sudan constituted a separated morphological class from golden jackals ranging in North Africa (Algeria, Tunisia and Libya) and East Africa (central Sudan and Ethiopia). The re-assessed range of the African wolf also supports a wide spectrum of habitats, ranging from Mediterranean, coastal and hilly areas (including hedged farmlands, scrublands, pinewoods and oak forests) in Algeria, to tropical, semi-arid climate zones including Sahelian savannahs in Senegal, and Sahelian massifs in Mali with as few as 100 mm annual rainfall [25] (Fig. 4).

The African wolf appeared as a distinct genetic entity (Figs. 2 and 3). Genetic distances with the other wolf lineages ranged between 1.9 and 4.3%, whereas they reached 4.5 to 9.3% between the African wolf and the different lineages of jackals (*CYTB*; Table 2). The uniqueness of the African wolf was reinforced by the fact that it had the highest level of haplotype and nucleotide diversity among gray wolf lineages, even exceeding that of the Holarctic wolves and dogs, and far greater than what was found for the Himalayan and Indian wolves (*CYTB* and CR; Table 3).

We showed that the African wolf contemporaneously coalesced with the lineage of Holarctic wolves and dogs, during the Late Pleistocene (Table 4; see [5]). The hypothesis of a recent dispersal of African wolves from North-East Africa thus appears unlikely, as it is also suggested by the presence of large ‘forms’ of jackals comparable to *C. l. lupaster* as early as the Middle to Late Pleistocene from North-West Africa [17]. The female effective population size (N_ef_) of the African wolf was 80,000, which fell within the range of *C. lupus/familiaris* (Table 5). Although lower than the average estimates for large Carnivores (felids and canids: 130,000–430,000) [26], such a value, together with the phylogenetic distinctiveness of the African wolf, its great level of genetic diversity and relatively ancient coalescence, argue for an ancient African lineage with, at least until recently, a fairly large female effective population size [27].

There was no apparent geographic structuring within the African wolves sampled, despite a 6,000 km wide geographic coverage (Fig. 3). Although our data are preliminary, we may insinuate that the great dispersal abilities of the gray wolf [5] also apply in its African lineage, and that potentially limiting factors such as habitat heterogeneity (notably vegetation cover) and prey availability [28] have not significantly impacted its population structure.

The newly expanded distribution of the African wolf poses the question of how such a large carnivore has gone undetected so long throughout its range. Although large wolf-like animals have been recorded from North Africa since Aristotle and again from the XIX^th^ century [9], mammalogists have generally considered that two forms of golden jackals (including the larger, Egyptian jackal ‘*lupaster*’) co-occurred in this region. The non-recognition of the African wolf probably is a result of poorly established morphological differences; the African wolf is larger than the golden jackal, but their size may overlap [9]. We provide here for the first time a morphological and behavioral diagnosis for the two entities on the basis of *in situ* observations (CB) of seven African wolves and c. 60 African golden jackals at the Senegalese site. *Canis l. lupaster* appears more heavily built, with a wider head, and a darker coat with a thick and longer hair cover; its tail is shorter, thickly furred and has a large and dark distal panache; it also has broader white breast-shield and stripes bordering the mouth, as well as larger and more round-shaped ears (Figs. 1 and 5). Focusing on behavioral traits, African wolves appear solitary and extremely shy, living at the periphery of family packs of golden jackals. Shepherds mentioned that the African wolf may hunt larger livestock such as sheeps, goats and even cows, whereas the golden jackal was only observed preying on lambs. The sole observed interactions between the two *Canis* were harassment and fighting by the African wolf for dead carcasses used by golden jackals, the latter inevitably abandoning their food to the former (Fig. 5).

Despite obvious morphological and behavioral differences, our results eventually questioned the delineation between the African wolf and the golden jackal ‘entities’. The taxonomic assignment procedure (and the phylogenetic analysis) identified the two *C. aureus* from Senegal as belonging to the African wolf mitochondrial lineage, including the fourth generation-captive bred individual (Table 1). Contrary to the *Canis* sampled from Algeria and Mali, excellent photographic documentation from Senegal allowed us to ascertain that the sampled individuals belonged to two distinct phenotypes, corresponding to the African wolf and the golden jackal (Fig. 5). The first hypothesis for explaining these results is that hybridization occurs between the two *Canis*, at least in Senegal. Although not reported in the wild between *C. aureus* and *C. lupus*, hybridization commonly occurs among *Canis* species and may yield viable hybrids [20], [21], [23], [29]. Besides, the drastically different behaviors of the two ‘phenotypes’ observed in Senegal (see above) reinforce the idea that both represents genuine species for which a shared mtDNA lineage could have only originated via hybridization. However, if hybridization occurred between the African wolf and the golden jackal, we would expect to observe intermediate morphotypes, although adaptive variation correlated to hybridization in canids may conceal the expected ‘intermediateness’ of the hybrids [24]. Referring to the diagnosis proposed above for the African wolf, a range of ‘intermediate’ morphotypes could be observed at the Senegalese study site (Fig. 6). Unfortunately, the identification of intermediate individuals is rendered difficult by the lack of knowledge on morphological variation occurring in both *C. l. lupaster* and *C. aureus*. At this stage, we cannot consider that such observed variability comes into support of the hybridization hypothesis.

The second hypothesis that would explain the detection of *C. l. lupaster* mtDNA in *C. aureus* is that the African golden jackal in North and West Africa is just an eco-morphological variant within the African wolf lineage. It has been suggested that *C. aureus* from Africa was distinct and significantly larger than its Asian counterpart [12] because of character release following the absence of wolves on the African continent [30]. However, it is most likely that *C. l. lupaster* has been roaming in Africa since (at least) the Middle to Late Pleistocene, and that the African wolf and a cline of smaller morphotypes, traditionally defined as ‘golden jackals’, have been co-occurring in Africa since that period, without any clear morphological, temporal or ecological delineation [17]. At this stage of our investigations, both hypotheses (hybridization and eco-morphotype) remain plausible.

Finally, our study raises the question of the delineation among species of African jackals. The *CYTB* sequences representing in Genbank the three species of jackals did not cluster into monophyletic groups: neither among themselves (*C. mesomelas*: *CYTB* distance between haplogroups = 9.3%; *C. aureus*: 6.4%), nor with our newly produced sequences (*C. adustus*: 13.5%) (Fig. 3; Table 2). Our results may evidence cryptic diversity within African jackals that should deserve further investigations (although the problematic nature of this Genbank series of sequences has also been pointed out [13]). This situation is especially detrimental for mtDNA-based typing procedures applied to African canids, for which morphological distinction among species remains challenging [31], [32], [33]. Because morphological variation in jackals has been assessed only at a local scale, a better delineation of the diagnostic characters is needed (for instance, *C. adustus* from West Africa has a black tail tip instead of the ‘diagnostic’ white tail tip in East Africa [34]; CB and PP, pers. obs.).

Our study provides a new characterization of the African wolf through the first comprehensive genetic, geographic and phenotypic reassessment of the lineage. *Canis l. lupaster* clearly appears as a distinct, relatively ancient gray wolf lineage encompassing a range 6,000 km wide, stretching from Senegal to Egypt. Increasing the geographic and genetic coverage of the present investigation will be necessary to further characterize the delineation among African wolf and jackal species, the dynamics of gene flow within the African wolf, and the impact of potential hybridization with the golden jackal on the distribution and adaptive nature of wolf- and jackal-like phenotypes. Given that ‘jackal-like’ canids in Africa are regularly killed to protect livestock, it appears urgent to engage into a conservation strategy for the benefit of the African wolf.

## Materials and Methods

Observations of wolf-like canids (hereafter referred to as *Canis* sp.) living at the near periphery of packs of golden jackals (*Canis aureus*) were made in July 2011 by CB, twelve km east from Parc National du Djoudj, in northern Senegal. At this site, *Canis* sp. individuals were distinctively larger and darker than the golden jackals (Fig. 1). They also behaved differently, presenting solitary and shy demeanors (see Discussion). Hair samples were collected from a wolf-like individual at one of the observation sites. The samples were gathered at a specific resting site right after the animal left. We also collected hair samples from one golden jackal individual from the same observation site and a fourth generation-captive bred specimen originating from a wild-born Senegalese golden jackal pack (Zoo du Parc de Hann, Dakar). Six additional tissue samples of *Canis* sp. were obtained from the coastal region of northeastern Algeria and Adrar des Iforas, eastern Mali (Fig. 1). To improve the accuracy of phylogenetic assignment among African *Canis*, we also sampled tissues of side-striped jackals (*Canis adustus*) from western Africa (Table 1).

DNA from hair and tissue samples was extracted using a standard CTAB extraction protocol [35] and an ABI PRISM© 6100 Nucleic Acid PrepStation (Applied Biosystems, Carlsbad, CA) following manufacturer’s recommendations, respectively. Two mtDNA fragments from the cytochrome *b* (*CYTB*; 402 bp) and control region (CR; 287–337 bp) were amplified by PCR, using the primer pairs GVL14724-H15149 [36] and CR1F-CR2R [37], respectively. PCR amplification protocols followed Gaubert et al. [36]. PCR products were visualized on a 1.5% agarose gel, purified and directly sequenced in both directions on 3730xl DNA Analyzer 96-capillary sequencers (Applied Biosystems, Foster City, CA) at Genoscope (Evry, France) and Eurofins MWG Operon (Ebersberg, Germany).

All the sequences produced from this study were deposited in Genbank under the accession numbers JQ088650–JQ088665 and JQ088669–JQ088684 (Table 1).

Nucleotide sequence alignments were preliminarily reconstructed using Clustal X 2.1 [38] and completed with BioEdit 7.0.9 [39]. The taxonomic assignment of the newly generated sequences of African *Canis* was done using a Bayesian framework, assessing sequence assignment through the Statistical Assignment Package 1.0.6. (SAP; [40]). The latter statistically assesses the level of confidence of sequence assignment by using a Bayesian phylogenetic reconstruction among a set of similarity-selected nucleotide sequences [40]. Consequently, the accuracy of the method is greater when (i) the genetic and geographic diversity of the taxa under study is well represented in Genbank, and (ii) the genetic diversity is structured into well-supported clades. We determined the minimum identity parameter to be 0.9, which corresponds to the lower bound (90%) of the level of similarity among retrieved sequences.

We ran a phylogenetic analysis of the wolf-like clade using the Bayesian Markov Chain Monte Carlo (MCMC) procedure implemented in BEAST v. 1.6.2 [41]. *CYTB* and CR were arranged into a single ‘coherent’ segment, to which we applied the model HKY + I + Γ [42] as selected by jModelTest v. 0.1 with the AIC and BIC criteria [43]. The sample set was restricted to our generated sequences and a sub-sample of the wolf-like clade representatives for which this coherent segment (*CYTB* + CR) was covered in Genbank. We did not use outgroups since BEAST samples the root position (along with the rest of the tree nodes). We assumed a constant size coalescent model [41]. Operators were tuned manually after screening of effective sample size values from preliminary runs, to maximize their efficiency and obtain convergence in poorly estimated parameters [44]. Chain lengths were 100,000,000, sampled every 10,000 generations. Analyses were run twice independently. Log files were concatenated under LogCombiner 1.6.2 [44] with a final burn-in of 1,000. Convergence and stability of estimated parameters were checked using Tracer 1.5 [45].

In order to assess the jackal – wolf haplogroup relationships in a comprehensive framework, we built a median-joining network from the *CYTB* sequences representing *Canis lupus* and the three jackal species. We did not include the CR sequences in our network analysis because only one species of jackals was represented in GenBank (*Canis aureus* from Eurasia). All the *CYTB* sequences representing gray wolves and jackals that aligned with our *CYTB* fragment with a reasonable amount of nucleotide coverage (>75%) were downloaded from Genbank, resulting in datasets of 346 (*CYTB*) (alignment available upon request to the corresponding author). We used Network 4.6.0.0 (http://www.fluxus-engineering.com), with ε fixed to 0 in order to minimize alternative median networks.

We used MEGA5 [46] to calculate the *CYTB* mean distances among the main phylogenetic lineages within the wolf-like clade, using the Kimura 2-parameter model (K2P) in order to provide estimates referable to the framework of the Genetic Species Concept [47], [48]. All positions with less than 95% site coverage were eliminated before analysis. Standard error was estimated by a bootstrap procedure (500 replicates).

We used DnaSP 5.10 [49] to calculate the number of polymorphic sites (*S*), nucleotide diversity (π), number of haplotypes (h) and haplotype diversity (Hd) among the four main mtDNA lineages of *Canis lupus* found in our analyses (*lupus/familiaris, pallipes, chanco* and *lupaster*; see Results). Since DnaSP automatically considers missing data and indels as unique features, we removed nucleotide sequences and/or alignment blocks containing missing data and indels before analysis [36].

We calculated time to most recent common ancestor (TMRCA) in the four *Canis lupus* lineages independently, following the same analytical procedure in BEAST as described above. We used the rough evolutionary rate of 5.48% per million years estimated for the mitochondrial fragment encompassing *CYTB* to CR in canids [50], under a strict clock model. Given the straightforward calculation of this evolutionary rate, estimates derived from the latter should be regarded with some level of cautiousness.

We used LAMARC 2.1.6 [51] to estimate the parameter Θ in the four *Canis lupus* lineages with a coalescent, maximum likelihood approach using a Markov Chain Monte Carlo (MCMC) genealogy sampler. *CYTB* and CR were again arranged into a single ‘coherent’ segment. We used the F84 model of evolution [52] and ran two simultaneous searches twice, including 1,000,000 steps for the heating scheme representing the 10 initial chains and 20,000,000 steps for the two final chains (burn-in = 10,000 steps). Percentage profile likelihoods were used to calculate the 95% confidence interval for Θ. From the relationship Θ = 2N_ef_ *mu* (where N_ef_ is the effective population size of females and *mu* is the mutation rate expressed as number of mutations per site per generation), we derived N_ef_ from the evolutionary rate of Li et al. [50] (see above), fixing the generation time to three years [5].
